# Superior outcome of labial mucosal autograft over limbal allograft in the management of recurrent pterygium with symblepharon: a case report

**DOI:** 10.3389/fmed.2024.1452579

**Published:** 2024-10-23

**Authors:** Joo Youn Oh, Sang In Khwarg

**Affiliations:** ^1^Laboratory of Ocular Regenerative Medicine and Immunology, Biomedical Research Institute, Seoul National University Hospital, Seoul, Republic of Korea; ^2^Department of Ophthalmology, Seoul National University College of Medicine, Seoul, Republic of Korea

**Keywords:** labial mucosal autograft, limbal allograft, oral mucosal graft, pterygium, symblepharon

## Abstract

A 40-year-old woman visited our clinic for recurred pterygium and symblepharon in the right eye. She had a history of pterygium excision 8 years before. Over the course of 7 years, we performed pterygium excision combined initially with mitomycin C (MMC) application and conjunctival autograft. This was followed by three procedures using limbal allografts, MMC application, and amniotic membrane transplantation. All procedures were unsuccessful, resulting in aggressive recurrences of pterygial mass and symblepharon, extraocular movement limitation, corneal astigmatism, and decreased visual acuity. Ultimately, we applied a labial mucosal autograft after the recession of pterygial tissue. No complications were observed. Two and a half years postoperatively, the labial mucosal autograft was well-integrated into the conjunctival surface without symblepharon recurrence or abduction limitation. Corneal clarity was restored, and astigmatism was reduced, with no recurrence of pterygium. In conclusion, a labial mucosal autograft is a viable treatment option in complex cases of recalcitrantly recurrent pterygium with symblepharon.

## Introduction

The recurrence rate following primary pterygium surgery varies widely depending on patient demographics, pterygium morphology, and surgical techniques (1–3). Younger age and the fleshy appearance of pterygium are associated with higher rates of recurrence after excision. Among surgical techniques, conjunctival autograft is associated with a lower risk of recurrence (4). Our group previously reported a recurrence rate of 2.3% for primary pterygium following excision and free conjunctival autograft over an average follow-up period of 36.2 months (5). Similarly, other studies have reported recurrence rates of 0–9% for primary or recurrent pterygium treated with conjunctival or limbal–conjunctival autografts (6–9).

The surgical outcomes of recurrent pterygium are poor compared to those of primary pterygium (1). Fibrovascular tissue proliferation is more prominent in recurrent pterygium, leading to more significant recurrences of the pterygial mass after surgery (1). Moreover, recurrences of pterygium can cause severe conjunctival scarring and shortening, which may result in conjunctival insufficiency for further conjunctival autografting and symblepharon-induced extraocular movement (EOM) restriction. For the management of these cases, limbal allografts and mucous membrane autografts from other sites, such as oral or nasal mucosa, have been suggested (10–12).

In this study, we present a successful outcome of labial mucosal autograft in a patient with recalcitrantly recurrent pterygium and severe symblepharon despite repeated surgeries using conjunctival autograft, limbal allografts, mitomycin C (MMC) application, and amniotic membrane (AM) transplantation.

## Case presentation

**Figure 1:**
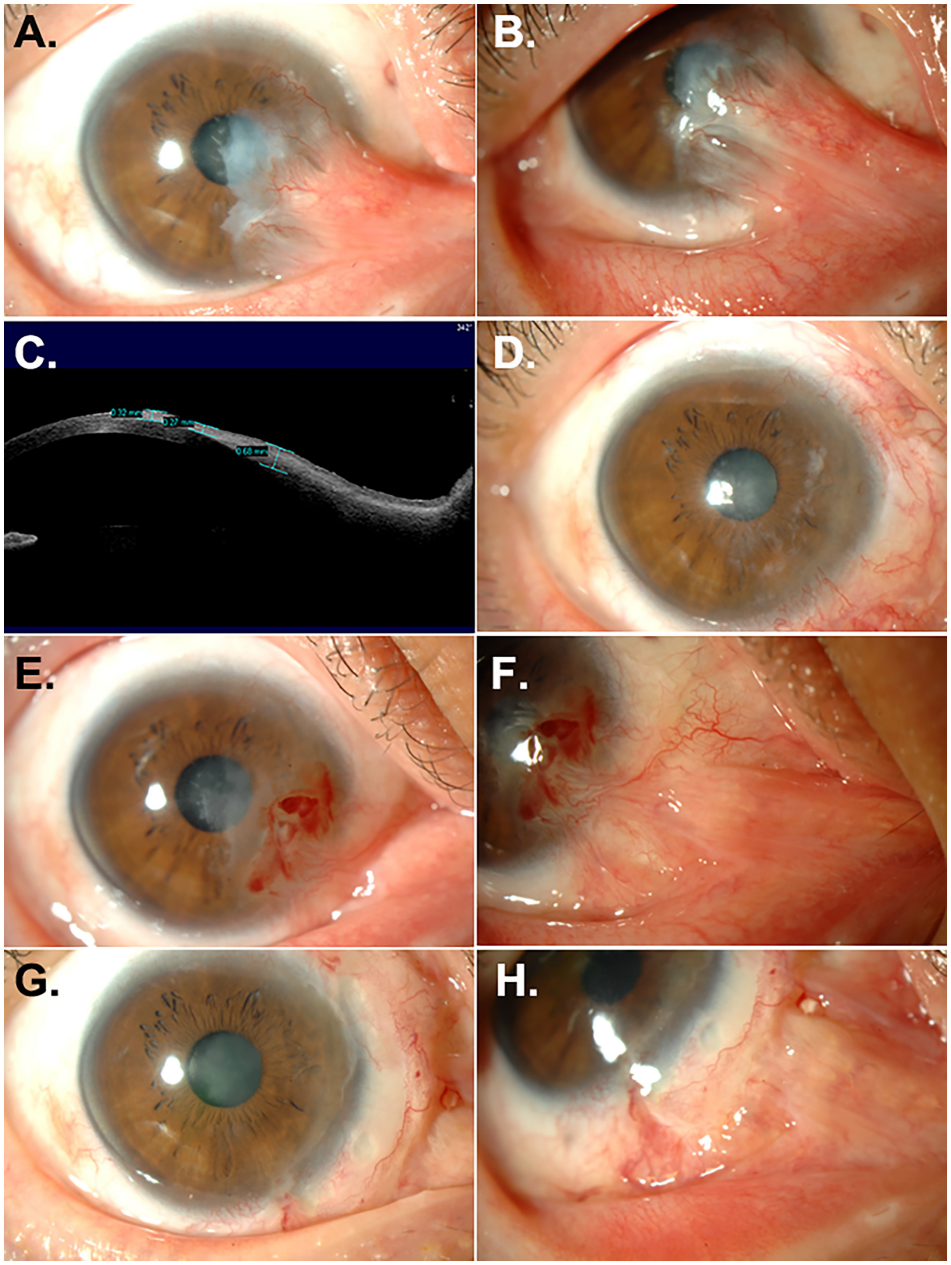
Recurrence of pterygium and symblepharon after treatment with conjunctival autograft. **(A, B)** Anterior segment photographs of a patient at presentation. A fleshy pterygial mass and symblepharon extending to the medial end of the lower lid were noted. **(C)** Anterior OCT at presentation. Up to 0.68 mm thick pterygial mass invaded the corneal surface. **(D)** One month after the first surgery using pterygium excision, symblepharolysis, intraoperative MMC application, and conjunctival autograft. **(E, F)** Six months after the first surgery. Fibrovascular ingrowth into the cornea causing 10.0 D of astigmatism and symblepharon recurrence was observed. **(G, H)** One month after a second surgery using pterygium excision, symblepharolysis, intraoperative MMC application, limbal allograft, and AM transplantation.

A 40-year-old woman presented to our clinic with a recurrent pterygium and symblepharon in her right eye, 8 years after a previous pterygium excision. Her best corrected visual acuity (BCVA) was 20/60 with +4.00 D −8.50 D × 176. The slit lamp biomicroscopic examination showed a fleshy pterygial mass with central corneal invasion and severe symblepharon extending to the medial end of the lower lid, resulting in inferior fornix obliteration and abduction limitation (Figures 1A, B). Anterior segment optical coherence tomography (OCT) examination demonstrated a pterygial mass invading the corneal surface, with a thickness measuring up to 0.68 mm (Figure 1C). For treatment, we performed pterygium excision, MMC application, and a free conjunctival autograft (#1). The pterygium, along with an additional 1 mm of corneal epithelium around the leading edge (13), was removed, and the symblepharon was released using a beaver blade and Westcott scissors. Several Weck-Cel sponges soaked in 0.2 mg/ml MMC were applied to the adjacent subconjunctival space for 1 min, followed by thorough irrigation with 50 ml of balanced salt solution (BSS). A free conjunctival graft, sized to match the conjunctival defect, was prepared by dissecting the superotemporal conjunctiva near the limbus from the underlying Tenon's capsule using Vannas scissors and secured to the pterygium excision site with 10-0 nylon interrupted sutures, ensuring that the limbal edge of the graft aligned with the limbal edge of the defect site. A bandage contact lens (BCL) was applied and left in place for 1 week. Levofloxacin 0.5%, prednisolone 1%, and autologous serum were topically administered for 3 weeks, and all sutures were removed. One month after surgery, the corneal surface was fully epithelialized, and the graft was well-adapted to the surrounding conjunctiva (Figure 1D). Corneal astigmatism was reduced, and BCVA improved to 20/20 with +1.00 D −2.00 D × 154.

Six months after surgery, a recurrence of pterygium and symblepharon was observed (Figures 1E, F), accompanied by severe astigmatism (+2.00 D −10.00 D × 149). We opted for surgery (#2) utilizing a limbal allograft due to insufficient conjunctiva for an autologous conjunctival graft. The limbal graft was obtained from a deceased donor cornea with a preservation time of ≤ 5 days and a donor age of ≤ 60 years (Eversight, Ann Arbor, MI) and was prepared by slicing the tissue into anterior and posterior lamellae using a sclerotome blade. The anterior lamella, including the conjunctival tissue, was used for grafting. After wide excision of the pterygium and fibrovascular tissue and lysis of symblepharon, several sponges soaked in 0.2 mg/ml of MMC were placed beneath the adjacent conjunctival edge for 1 min, followed by 50 mL of BSS irrigation. AM was placed over the bare sclera with the epithelial side up and secured with interrupted 8-0 vicryl sutures. The prepared limbal allograft was trimmed to cover the defect area and fixed atop the AM on the sclera with 10-0 nylon sutures. The conjunctival tissue from the donor graft was adapted to the surrounding conjunctiva with continuous 8-0 vicryl sutures. The graft and de-epithelialized cornea were covered with an overlay of AM using interrupted 8-0 vicryl sutures, and BCL was applied for 1 week. Postoperatively, oral cyclosporine (150 mg BID) and oral prednisolone (30 mg QD) were administered, in addition to topical levofloxacin 0.5%, prednisolone 1%, and autologous serum. Oral cyclosporine was maintained for 6 months, and oral prednisolone was tapered over 3 weeks. One month after the surgery, the graft was well-incorporated with full epithelialization of the graft and corneal surface, and the symblepharon was successfully released (Figures 1G, H).

**Figure 2:**
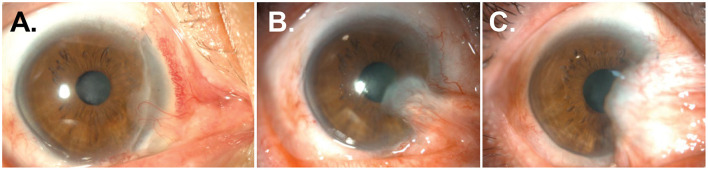
Recurrences of pterygium and symblepharon after repeated surgeries using limbal allografts and AM transplantation. **(A, B)** Anterior segment photographs of the patient 6 **(A)** and 30 months **(B)** after a second surgery. Pterygial mass and symblepharon started to reform and overgrew onto the previous limbal graft and corneal surface progressively. **(C)** Anterior segment photographs of the patient 6 months after a fourth surgery.

**Figure 3:**
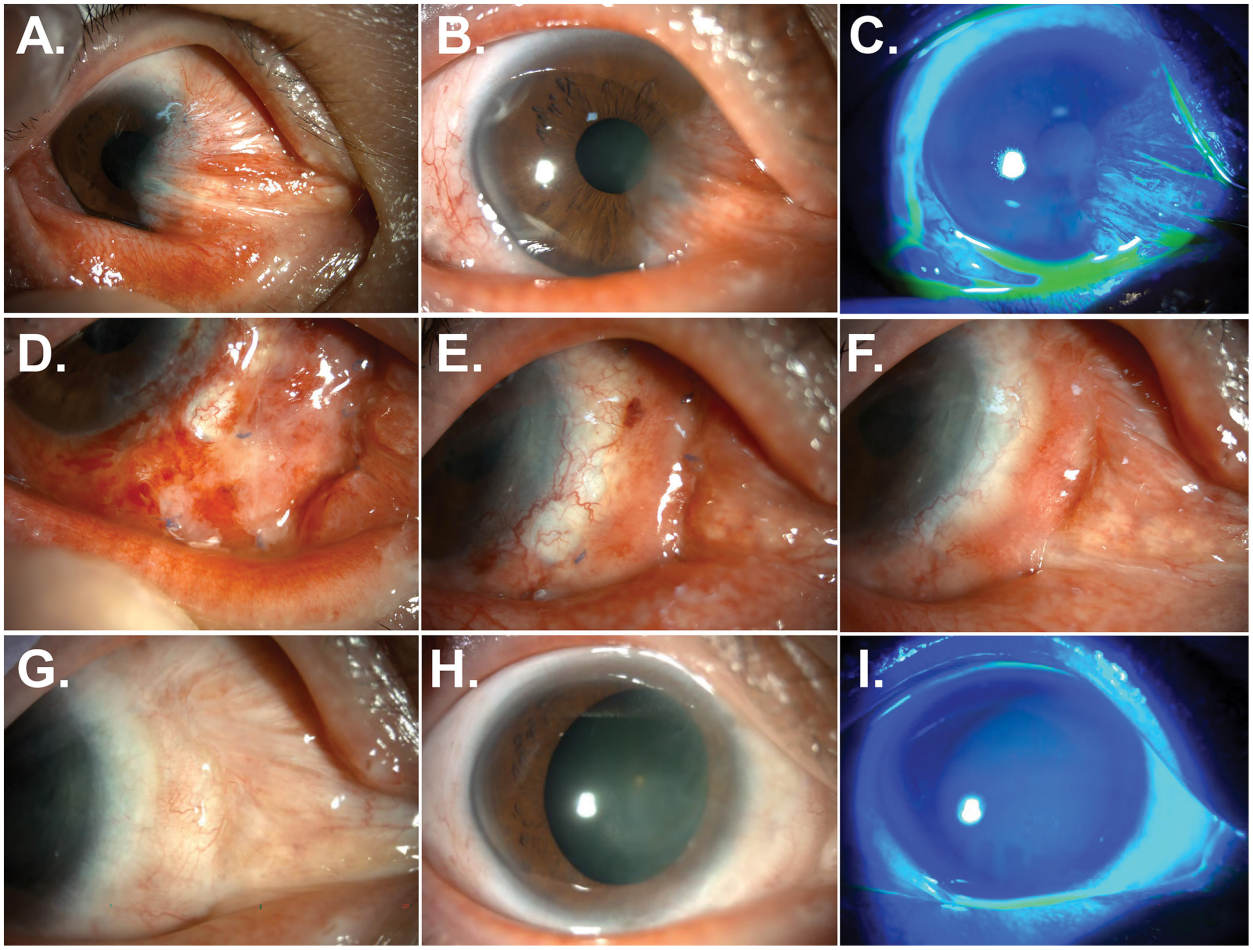
Resolution of pterygium and symblepharon after treatment with labial mucosal autograft. **(A–C)** Anterior segment photographs of the patient 12 months after a fourth surgery. A recalcitrant recurrence of pterygium and symblepharon was observed. **(D–F)** Anterior segment photographs of the patient at 1 week **(D)**, 3 weeks **(E)**, and 3 months **(F)** after a fifth surgery using labial mucosal autograft. **(G–I)** Anterior segment photographs without and with fluorescein staining at 30 months after labial mucosal autograft. Pterygium and symblepharon did not recur, and a smooth and stable ocular surface was achieved.

**Figure 4:**
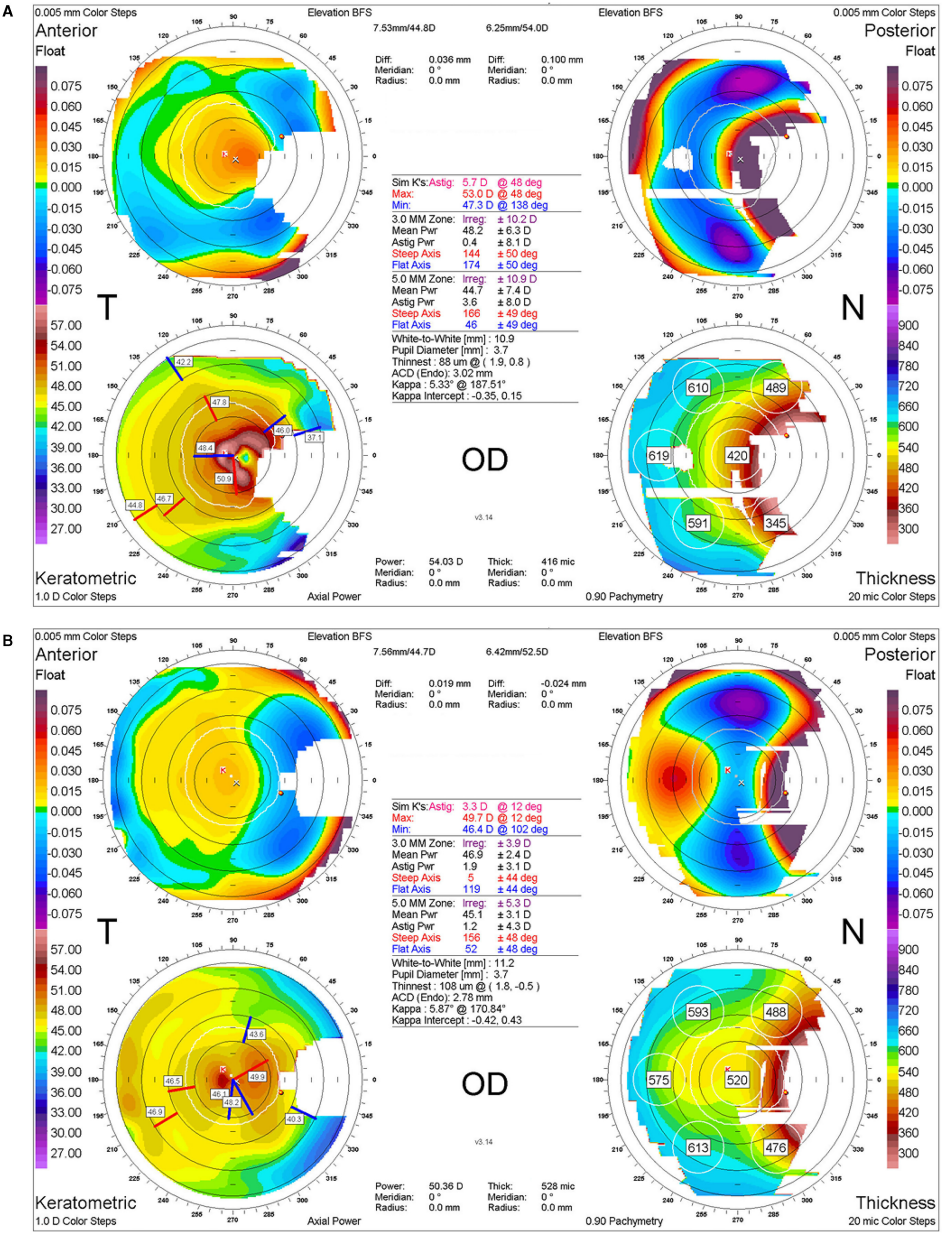
Corneal topography before and after labial mucosal autograft. **(A)** Corneal topographic image at the time of recurrence 3 years after a third surgery. **(B)** Corneal topographic image 2 years after a fifth surgery using labial mucosal autograft, indicating a significant reduction in corneal astigmatism and irregularities with the resolution of pterygium.

Six months after surgery, pterygium and symblepharon began to reform and gradually progressed (Figure 2A). Two and a half years after surgery, the recurrent pterygium and symblepharon overgrew their original size, occluding the visual axis and causing EOM limitation on the right gaze (Figure 2B). Thus, the surgery (#3) was performed using a limbal allograft, intraoperative MMC application, and AM transplantation. Three years later, a severe recurrence of pterygium and symblepharon occurred, leading to the induction of 5.7 D of irregular corneal astigmatism (Figure 4A), necessitating another surgery (#4) using the same technique. Six months after surgery, pterygium and symblepharon recurred aggressively (Figure 2C) and progressed rapidly (Figures 3A–C). Given the multiple and accelerated recurrences after repeated surgeries with limbal allografts, we decided to adopt a labial mucosal autograft (#5). The leading edge of pterygium overlying the cornea was lifted and excised using a beaver blade and Westcott scissors. The remaining pterygial tissue was then medially dissected from the underlying sclera until reaching the tendinous area of the medial rectus muscle. The lateral edge of the pterygial tissue was recessed medially and secured to the area around the insertion of the medial rectus muscle with 8-0 polyglactin interrupted sutures. A free labial mucosal graft (4 × 17 mm), tailored to fit the conjunctival defect, was harvested from the lower lip and placed over the bare sclera to cover the conjunctival defect, leaving a perilimbal 1.5 mm area exposed. It was then secured to the sclera with an 8-0 polyglactin interrupted sutures (Figure 3D). A BCL was applied and remained in place for 1 week. Levofloxacin 0.5% and prednisolone 1% were topically administered for 8 weeks. No intraoperative or postoperative complications were observed. Three weeks after surgery, the corneal surface was fully epithelialized, and the graft was well-adapted to the surrounding conjunctiva (Figures 3E, F).

Two and a half years after surgery, the labial graft was perfectly integrated into the ocular surface, resulting in a smooth ocular surface and an excellent cosmetic outcome (Figures 3G–I). Neither pterygium nor symblepharon recurred. Corneal astigmatism was reduced, and regular astigmatism was maintained at 3.3 D (Figure 4B).

## Discussion

Ocular surface reconstruction after pterygium excision is crucial for preventing disease recurrence. Transplantation of the patient's healthy conjunctiva is the ideal option for the replacement of conjunctival defects. However, in cases of conjunctival insufficiency, alternative graft materials such as amniotic membrane, allogeneic limbal tissue, autologous nasal mucosa, and oral mucosa (e.g., labial, buccal, and hard palate mucosa) are necessary for effective treatment and prevention of the disease. In this study, we report a superior outcome of labial mucosal autograft compared to conjunctival autograft, limbal allograft, and AM transplantation for the treatment of refractory pterygium combined with symblepharon.

Oral mucosal autografting has been applied to various ophthalmic indications, including the management of restricted socket syndrome, cicatricial ocular surface diseases, conjunctival defects after glaucoma or retinal surgery, and ocular surface and fornix reconstruction following tumor or symblepharon resection (12, 14, 15). Oral mucosa, with biological properties similar to conjunctiva, offers many advantages as a grafting material. It is easily accessible, widely available for sufficient sizes and repeated harvesting, and highly stable and well-tolerated when transplanted to the ocular surface. Moreover, oral mucosal autograft does not carry the risk of allogeneic immune rejection, thereby eliminating the need for long-term corticosteroid or immunosuppressive therapy. This reduces risks associated with limbal allograft, such as immune rejection and side effects related to steroid or immunosuppressant use. In fact, in our patient, limbal allografts failed three times, leading to multiple recurrences of pterygium and symblepharon. Given that we used healthy donor tissues (with a preservation time of ≤ 5 days and a donor age of ≤ 60 years) for the limbal allografts, which presumably contained a substantial number of limbal stem cells, it is possible that alloimmune rejection contributed to the failure of the grafts in our patient. To prevent rejection, our patient received oral cyclosporine for 6 months and oral prednisolone for 3 weeks, in addition to topical corticosteroids, after each limbal allografting surgery. Unfortunately, the prolonged use of oral cyclosporine and topical corticosteroids led to the development of hypertension and posterior capsular cataract in our patient. Ultimately, the use of oral mucosal autograft—specifically labial mucosa due to its thinner nature—resulted in excellent cosmetic, anatomical, and functional outcomes in our case, with medication required for no longer than 4 weeks postoperatively. The main disadvantage of oral mucosa graft is its inability to provide lubrication to the ocular surface because of the lack of goblet cells. Therefore, the efficacy of oral mucosal graft may be limited in patients with severe dry eye syndrome or cicatricial diseases such as Stevens–Johnson syndrome, chemical burns, or ocular pemphigoid (15).

In conclusion, we propose that labial mucosal autograft may be a safe and effective option for recurrent cases of severe pterygium and symblepharon unresponsive to other treatments, such as conjunctival autograft, AM transplantation, and limbal allografts.

## Data Availability

The original contributions presented in the study are included in the article/supplementary material, further inquiries can be directed to the corresponding author.
